# Prediction of Iron Deficiency Anemia in Third Trimester of Pregnancy Based on Data in the First Trimester: A Prospective Cohort Study in a High-Income Country

**DOI:** 10.3390/nu14194091

**Published:** 2022-10-01

**Authors:** Anne-Sophie Resseguier, Candy Guiguet-Auclair, Anne Debost-Legrand, Anne-Françoise Serre-Sapin, Laurent Gerbaud, Françoise Vendittelli, Marc Ruivard

**Affiliations:** 1 Université Clermont Auvergne, CHU Clermont-Ferrand, CNRS, Institut Pascal, F-63000 Clermont-Ferrand, France; 2 Internal Medicine Department, CHU Clermont-Ferrand, F-63000 Clermont-Ferrand, France; 3 Public Health Department, CHU Clermont-Ferrand, F-63000 Clermont-Ferrand, France; 4 Réseau de Santé Périnatale d’Auvergne, CHU Clermont-Ferrand, F-63000 Clermont-Ferrand, France; 5 Hematology Department, CHU Clermont-Ferrand, F-63000 Clermont-Ferrand, France

**Keywords:** iron, anemia, pregnancy, hemoglobin

## Abstract

Background: Systematic iron supplementation may be harmful in pregnant women with non-depleted iron. Our objectives were to estimate the prevalence of anemia at the third trimester of pregnancy (T3) and to identify the parameters at the first trimester (T1), which best predict anemia at T3. Methods: This prospective cohort study in France included pregnant women at T1 without non-iron deficiency anemia. Clinical and social characteristics, health-related quality of life, blood count, and a frozen blood sample were collected at T1 and/or T3. Secondly, a matched nested case–control study was built for women with anemia at T3 but not at T1. Multivariate analyses and ROC curves were used to identify the best predictive parameter(s) of anemia at T3. Results: The prevalence of anemia at T3 in the cohort (629 women) was 21.9% (95% CI 18.7–25.2%). In the matched nested case–control study (256 women), hemoglobin (Hb), serum ferritin (SF) and the SF/soluble transferrin receptor ratio at T1 were predictive of anemia at T3 (*p* < 0.001); however, clinical and social characteristics, as serum hepcidin were not. In multivariate analyses, Hb at T1 was the best predictive biomarker of anemia at T3 with a cut-off value of 120 g/L (specificity 87.5%). Conclusions: The prevalence of anemia at the end of pregnancy remained high in a High-Income Country. Clinical, social, and biochemical parameters did not seem useful to predict anemia at T3 and could not guide iron supplementation. We suggest systematically performing a simple blood count in the first trimester of pregnancy and offering oral iron supplementation for women with Hb < 120 g/L.

## 1. Introduction

Iron deficiency anemia (IDA) is very common in pregnancy, particularly in the third trimester, due to the increase in iron requirement by the fetus. The consequences of IDA during pregnancy can be serious for the mother: decreased cognitive and working capacities, depression, and susceptibility to infections [1]. Iron deficiency is the leading cause of anemia during pregnancy. For newborns, IDA in the mother is associated with neurological disturbances and long-term sequelae such as memory disorders, autism, and schizophrenia [2]. In High-Income Countries, the prevalence of anemia during pregnancy is only 22% [3], and routine iron supplementation is not recommended [1]. Moreover, systematic iron supplementation could be harmful in non-anemic women: hepcidin (Hepc) is decreased during pregnancy, leading to better iron bioavailability and thus to the risk of stimulating erythropoiesis and an increase in hemoglobin (Hb) concentration. This hemoconcentration is associated with low birth weight and premature deliveries [4]. Finally, both maternal Hb < 110 g/L or >130 g/L are associated with poor birth and adverse maternal health outcomes [5]. 

Therefore, we need to identify early in pregnancy women at risk of developing iron deficiency anemia in late pregnancy in order to better target iron supplementation. In clinical practice, serum ferritin (SF) is the most reliable marker to diagnose iron deficiency in pregnancy [6]. Other biomarkers such as Hepc, soluble transferrin receptor (STfR) and the SF/STfR ratio may have better sensitivity and/or specificity [7,8], but studies during pregnancy are lacking. The two main objectives of this study were: (i) to estimate the prevalence of anemia at the end of pregnancy among women followed-up in a reference maternity unit in France and (ii) to identify what were the clinical factors and biomarkers at the first trimester of pregnancy (including Hb, mean corpuscular volume (MCV), SF, STfR, SF/STfR ratio and Hepc) that best predicted anemia at the end of pregnancy.

## 2. Materials and Methods

### 2.1. Study Design, Setting, and Participants

A prospective non-interventional single-center cohort study was conducted during one year in France. The study was approved by the French Medical Ethics Committee for the Protection of Individuals Southeast VI (CPP Sud-Est VI, no. AU1184, 16 September 2015) and registered on ClinicalTrials.gov (NCT03176147). All the women provided written consent to participate in the study after being informed in detail about the study procedures. 

**Figure 1:**
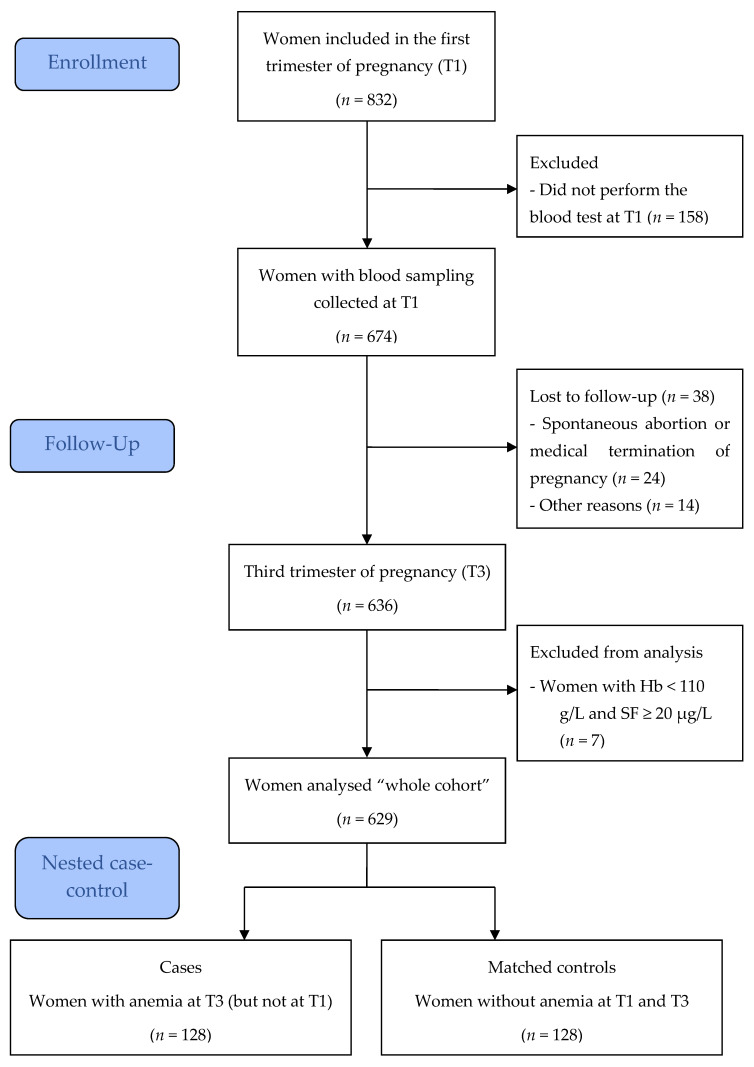
Flow diagram of participants.

Whole cohort: Pregnant women who consulted the maternity department of Clermont-Ferrand University Hospital for an ultrasonography examination of the fetus between 9 weeks and 11 weeks plus 6 days of gestation, called “first trimester ultrasonography”, and routinely covered by the French public health insurance system were invited to participate in this study. According to French guidelines, women at risk of anemia have a free Hb screening in the first trimester of pregnancy (T1) [9] but a systematic iron supplementation is not recommended [10]. Women under 15 years of age were excluded due to the high prevalence of iron deficiency in adolescent pregnant women, warranting oral iron supplementation. Other exclusion criteria included: not performing the blood test at T1, anemia without iron deficiency (Hb < 110 g/L and SF ≥ 20 µg/L), spontaneous abortion or medical termination of pregnancy before the third trimester of pregnancy (T3), and the lost to follow-up at T3 (Figure 1).

Matched nested case–control study: A sub-group of the whole cohort was subsequently included in a secondary analysis. Each woman without anemia at T1 but with anemia at T3 was matched to one woman without anemia in T1 and T3, according to age (±5 years), body mass index before pregnancy (BMI < or ≥30 kg/m^2^), and the nearest date of inclusion in the study. We did not choose additional matching criteria such as parity, which can be an explanatory criterion of iron deficiency anemia.

After inclusion, a blood sample was taken for the blood count, and an additional tube was frozen. SF was systematically performed for women with anemia (defined as Hb < 110 g/L) according to international recommendations [6,8]. We subsequently excluded women with Hb < 110 g/L and SF ≥ 20 µg/L because they probably had non-iron deficiency anemia. For women with Hb < 110 g/L and SF < 20 µg/L, oral iron supplementation was systematically offered with one of the iron medications reimbursed by the French public health insurance system and according to WHO recommendations: iron sulfate (two tablets per day corresponding to 160 mg of elemental iron) or iron fumarate (two tablets per day corresponding to 132 mg of elemental iron per day). For women with Hb ≥ 110 g/L, we did not recommend iron supplementation and instructed them not to take any oral supplementation containing iron. 

A second blood count was systematically performed during a visit scheduled at T3 between 31 and 34 weeks of gestation. 

### 2.2. Data Collection

The blood samples collected at T1 were used to determine Hb, mean corpuscular volume (MCV), mean corpuscular hemoglobin concentration (MCHC), red cell distribution width (RDW), and reticulocyte hemoglobin content (RHC). The blood samples frozen at T1 were used to determine serum ferritin (SF), serum hepcidin (Hepc), and serum transferrin receptor (STfR) for the women included in the case–control study. Ferritin was measured by chemiluminescence using a Dimension Vista analyzer from Siemens USA. STfR and Hepc were measured using ELISA methods (R&D systems USA and Peninsula Laboratories International USA, respectively). As the public financial support of this study was not sufficient to determine the biomarkers of the whole cohort, a matched nested case–control was used. 

Clinical and social characteristics were collected at T1, including the use of “Universal Medical Coverage” (CMU, free medical coverage for people with very low incomes in France, allowing them to benefit from all medical care free of charge) and health-related quality of life (HRQoL) and social deprivation, using the MOS SF-36 [11] and EPICES [12] self-administered questionnaires, respectively. MOS SF-36 consists of 36 items assigned to 8 multi-item scales: Physical functioning, Physical role, Mental health, Emotional role, Social functioning, Bodily pain, Vitality, and General Health. For each scale, a score between 0 and 100 is obtained, with higher values indicating better HRQoL. It showed lower quality of life for women with lower iron levels [13]. The EPICES index is composed of 11 items regarding material, psychosocial, and social problems. The EPICES score ranges from 0 to 100 (higher scores showing higher deprivation) with a validated deprivation cut-off of 30.17 (deprived ≥ 30.17, non-deprived < 30.17). 

Clinical characteristics at T3 and delivery information were collected: systolic and diastolic blood pressure, hypertension, term of delivery, and birth weight of newborns. The blood samples collected at T3 were used to determine Hb, MCV, and MCHC.

### 2.3. Statistical Analysis

The sample size was statistically determined to estimate a prevalence of anemia at T3 of 10%, considering the lowest value found in the literature in High-Income Countries [2], with a precision of 2% and an alpha value of 0.05. The number of women needed at T1 was 865. Given an anticipated dropout rate of 10%, the sample size was determined as 952 women. 

Continuous variables were expressed as medians and interquartile ranges; categorical variables were expressed as numbers and percentages. 

The prevalence of anemia at T1 and T3 defined as Hb < 110 g/L, the prevalence of hemoconcentration at T3 defined as Hb > 135 d/L and the 95% confidence intervals (95% CIs) were calculated.

For the nested case–control study, women with anemia at T3 were compared to matched women without anemia at T3 using Wilcoxon signed-rank tests for continuous variables, McNemar tests for binary variables, and Cochran’s Q tests for categorical variables. 

Conditional logistic regressions were performed to investigate which sociodemographic and clinical characteristics or biomarkers at T1 were predictive factors of anemia at T3 in the matched nested case–control sample, with a forward selection if needed. We included independent variables significant in the bivariate analyses at *p* < 0.20. Unadjusted and adjusted odds-ratios (ORs) and their 95% CIs were calculated. 

For the most predictive biomarker at T1 identified in the conditional logistic regressions, we estimated the area under the receiver operating characteristic (ROC) curve (AUC). The AUC ranges from 0.5 to 1, where higher values indicate better accuracy. Different thresholds were tested for this biomarker to find the optimal positive predictive value using the matched case–control sample. Sensitivity, specificity, and negative predictive value were also estimated. We conducted sensitivity analyses using the whole cohort sample excluding women with anemia at T1 if this biomarker was collected for the whole cohort and not only for the matched nested case–control sample. 

Statistical analyses were performed with SAS software (version v9.4, SAS Institute, Cary, NC, USA, 2002-20012) and conducted at a two-sided alpha = 0.05 significance level.

## 3. Results

Among the pregnant women included, 81.0% performed the blood test in T1 (Figure 1). Clinical data and blood samples were collected at T3 for 94.4%. After the exclusion of women with Hb < 110 g/L and SF ≥ 20 µg/L, 629 pregnant women were included in the final analysis (whole cohort). 

Table 1 describes the clinical and social characteristics of the pregnant women of the whole cohort at T1, Table 2 their clinical characteristics and biomarkers at T3, and Table 3 the results of their T1 blood count tests. 

The prevalence of anemia at T3 was 21.9% (95% CI 18.7–25.2%). Among these 138 women with anemia, 10 (7.2%) already had anemia at T1. The prevalence of anemia in the whole cohort at T1 was 3.2% (95% CI 1.8–4.6%). The prevalence of hemoconcentration at T3 was 6.2% (95% CI 4.3–8.1%). Among the 629 pregnant women of the whole cohort, 481 (76.5%) did not have anemia at T1 or T3, 10 (1.6%) had anemia at T1 but not at T3, 10 (1.6%) had anemia at T1 and T3, and 128 (20.3%) did not have anemia at T1 but had anemia at T3. 

These 128 women without anemia at T1 and with anemia at T3 were defined as the cases of the matched nested case–control study. These cases were matched with 128 women that did not have anemia at T1 or T3 (controls). The social and clinical characteristics and the biomarkers of these 256 women at T1 and at T3 are presented in Table 1, Table 2 and Table 3, respectively. 

All biomarkers at T1 were significantly different between cases and controls, except RDW, Hepc, and the ratio Hepc/SF (Table 3). Hemoglobin, serum ferritin, and ratio SF/STfR of women with anemia at T3 were significantly lower than in controls (all *p* < 0.001). MCV, MCHC, and RHC were also lower in women with anemia at T3 than in controls with lower significant *p* values. Women with anemia at T3 had significantly higher STfR values than controls.

Two conditional logistic regressions were performed to investigate the predictive factors at T1 of anemia at T3: model 1 with SF and STfR among the independent variables, model 2 with the SF/STfR ratio. The results of the multivariate analyses, adjusted for parity and educational level, are described in Table 4. In the models without variable selection method, Hb was the factor the associated most significantly with anemia at T3. Using a forward selection, only Hb at T1 were entered in the two models with an adjusted OR of 0.41 (95% CI 0.26–0.65). 

The ROC curves were therefore estimated with Hb for the matched case–control sample (*n* = 256) and for the whole cohort, excluding the 20 women with anemia at T1 (*n* = 609). The AUC were 0.70 (95% CI 0.63–0.76) and 0.68 (95% CI 0.63–0.73), respectively. In the matched nested case–control sample, the best positive predictive value for anemia at the end of pregnancy was 74.6% (95% CI 63.9–85.4%) for an Hb value < 120 g/L at T1. For this cut-off value, specificity was 87.5% (95% CI 81.8–93.2%), sensitivity 36.7% (95% CI 28.4–45.1%), and negative predictive value 58.0% (95% CI 51.1–65.0%). Using the same cut-off in the whole cohort (Hb < 120 g/L), the positive predictive value was 83.6% (95% CI 80.3–86.8%), specificity 85.7% (95% CI 82.5–88.8%), sensitivity 36.7% (95% CI 28.4–45.1%), and negative predictive value 40.5% (95% CI 31.6–49.5%). For the 81 women with anemia at T3 and Hb of 120 g/L or more at T1, the median SF at T1 was 26 µg/L (interquartile range 13–50). 

## 4. Discussion

In this prospective cohort study conducted in France, we found that clinical and social parameters were not helpful to predict anemia at T3. On the contrary, Hb less than 120 g/L at T1 had a positive predictive value of more than 80% for the whole cohort and a specificity above 85% to predict anemia at T3. This optimal cut-off value of 12 g/dL for Hb was higher than 1 g/dL than the recommended value defining anemia at T1 [3,5]. Other biomarkers obtained at T1, including SF, Hepc, and STfR, did not reach significance in the multivariate analysis. Moreover, the cost of each of these biochemical parameters was higher than the determination of a blood count. Interestingly, in a recent study [14], Noshiro K et al. found quite similar results. In their study conducted in Japan, first trimester Hb levels were significantly better predictors of anemia at T3 than the indices of iron metabolism, including SF, serum iron and transferrin saturation, with an optimal cut-off value of an Hb level at 12.6 g/dL at T1. Like Noshiro et al. [14], we suggest that Hb, which can be measured economically, could be an important indicator in clinical practice for predicting anemia at T3.

Why Hepc and the SF/Hepc ratio obtained at T1 did not predict anemia at T3 is unclear. Hepc is physiologically lowered in all pregnant women to increase dietary iron absorption, which is essential for the fetus [7]. Hepc would therefore indicate an increase in iron requirements and not really an iron deficient status, which could explain its poor predictive value for anemia at the end of pregnancy. It has been demonstrated in pregnant women that Hepc is a marker of iron or vitamin C food intake [15]. We did not collect nutritional data in our study, and it is possible that diet could have influenced Hepc levels. 

The prevalence of IDA was low (3.2%) at T1 and similar to that of non-pregnant women of reproductive age in France [16]. The prevalence of anemia increased dramatically during pregnancy to reach 21.9% at T3 despite iron supplementation at T1 of all women with IDA at T1. These results were expected in High-Income Countries [2,14,17,18]. 

A recent guideline [1] recommends iron supplementation or the determination of SF in multiparous women (more than three prior pregnancies) or in twin pregnancy, but these clinical data were not predictive of anemia at the end of pregnancy in our study. Our results do not warrant systematic iron supplementation in pregnant women of High-Incomes Countries; however, a blood count should be performed at T1. If the Hb is less than 110 g/L, an SF count should be performed to confirm IDA, but if the Hb value is between 110 and 120 g/L, iron supplementation could be provided. We emphasize that the situation is completely different for pregnant women in Low-Middle-Income Countries, where supplementation is recommended [3]: systematic iron or folic-iron supplementation reduces the risk of anemia at the end of pregnancy by about 45% and the risk of having a low-birth-weight baby by more than 10% [19].

In our study, the number of subjects needed was not reached. Nevertheless, the prevalence of anemia at T3 (21.9%) was higher than estimated (10%), and our sample size allowed us to estimate the prevalence of anemia at T3 with a precision of 3.2%, which was very close to our objective of 2%. Transferrin saturation (TfS) was not performed because we determined SF, which demonstrated better efficacy for diagnosing IDA in pregnancy [6,20]. Furthermore, the study of Noshiro et al. [14] demonstrated that the sensitivity of TfS is only 17% in predicting anemia in the third trimester, which validated our choice a posteriori. We decided to devote the budget of the study to more original and less studied parameters, Hepc and STfR, with the calculation of the SF/STfR ratio. In our study, SF and SF/STfR performed at T1 were predictive of anemia at T3 but were less effective than a “simple” blood count, with a lower cost. However, it cannot be ruled out that women with an Hb higher than 110 g/L at the first trimester and an SF less than 20 µg/L received iron supplementation. Hb seems to be a good diagnostic test but should not be considered as a screening test due to its poor sensitivity. Indeed, more than 60% of women with anemia at T3 have Hb > 120 g/L at T1 with normal iron stores. For these women, only nutritional recommendations can be offered.

## 5. Conclusions

In this prospective cohort study, we found that an Hb value lower than 120 g/L in the first trimester of pregnancy is a good positive predictive value with very good specificity to predict anemia at the end of pregnancy. In High-Income Countries, we suggest systematically performing a blood count in the first trimester of pregnancy and offering oral iron supplementation for women with an Hb lower than 120 g/L. However, this recommendation should be assessed in a controlled trial in order to confirm the clinical benefit for the mother and the child at birth and during the development of the child. 

## Figures and Tables

**Table 1:** Clinical and social characteristics at first trimester of pregnancy (T1) of pregnant women of the whole cohort, women with anemia at third trimester of pregnancy (T3) (cases) and women without anemia at T3 (controls).

Characteristics at T1	Whole Cohort	Women with Anemia at T3 (Cases)	Matched Women without Anemia at T3 (Controls)	*p*-Value
No. of women	629	128	128	
Age, year, median (IQR)	30.0 (27.0–33.0)	29.0 (25.5–33.5)	30.0 (27.0–33.0)	0.071
Age, year, *n* (%)				0.655
<20	12 (1.9)	3 (2.3)	2 (1.6)	
20–34	491 (78.1)	99 (77.3)	101 (78.9)	
≥35	126 (20.0)	26 (20.3)	25 (19.5)	
Education level, *n* (%)				0.121
Lower than high school	139 (25.2)	33 (31.1)	22 (20.8)	
High school	106 (19.2)	19 (17.9)	19 (17.9)	
Higher education	307 (55.6)	54 (50.9)	65 (61.3)	
Life status, *n* (%)				**0.039**
Live alone	45 (7.9)	10 (9.1)	2 (1.8)	
Live in couple	523 (92.1)	100 (90.9)	108 (98.2)	
Professional activity, *n* (%)	374 (65.8)	63 (56.8)	76 (68.5)	0.105
Current smoking, *n* (%)	113 (20.2)	18 (17.0)	21 (19.8)	0.711
BMI before pregnancy, kg/m^2^, median (IQR)				0.152
Underweight (<18.5)	52 (9.0)	13 (11.3)	8 (7.0)	
Normal weight (18.5 to <25)	346 (59.6)	64 (55.7)	75 (65.2)	
Overweight (≥25)	183 (31.5)	38 (33.0)	32 (27.8)	
Parity, *n* (%)				0.279
0	108 (18.6)	23 (19.7)	19 (16.2)	
1	272 (46.7)	49 (41.9)	61 (52.1)	
2	132 (22.7)	31 (26.5)	27 (23.1)	
≥3	70 (12.0)	14 (12.0)	10 (8.5)	
Twin pregnancy, *n* (%)	8 (1.4)	2 (1.7)	2 (1.7)	1.000
EPICES score ^1^, median (IQR)	14.8 (7.1–29.0)	18.3 (13.6–38.5)	14.8 (6.5–26.0)	**0.024**
Deprivation ^2^, *n* (%)	84 (23.5)	13 (35.1)	8 (21.6)	0.332
Very low incomes (under CMU), *n* (%)	92 (16.0)	25 (21.9)	10 (8.8)	**0.011**
MOS SF-36 scores ^3^, median (IQR)				
Physical functioning	90.0 (70.7–95.0)	85.0 (65.0–100)	90.0 (75.0–95.0)	0.643
Role physical	62.5 (43.8–81.3)	62.5 (43.8–81.3)	62.5 (50.0–75.0)	0.847
Bodily pain	67.5 (45.0–90.0)	57.5 (45.0–90.0)	77.5 (57.5–90.0)	0.070
Vitality	40.0 (25.0–50.0)	40.0 (25.0–50.0)	40.0 (25.0–50.0)	0.360
Mental health	68.0 (56.0–80.0)	68.0 (52.0–80.0)	72.0 (56.0–84.0)	0.315
Role emotional	83.3 (58.3–100)	83.3 (58.3–100)	83.3 (58.3–100)	0.833
Social functioning	75.0 (62.5–87.5)	75.0 (62.5–87.5)	75.0 (62.5–100)	0.907
General health	70.0 (60.0–80.0)	70.0 (60.0–80.0)	70.0 (55.0–85.0)	0.820

Significant *p*-Values are reported in bold text (*p* < 0.05). T1: first trimester of pregnancy; T3: third trimester of pregnancy; IQR: interquartile range; BMI: body mass index; CMU: universal medical coverage. ^1^ EPICES was completed by 357 women in the whole cohort and by 37 women in case and matched-control groups. ^2^ Defined as EPICES score ≥ 30.17. ^3^ MOS SF-36 was completed by 364 women in the whole cohort and by 39 women in case and matched-control groups.

**Table 2:** Clinical characteristics and biomarkers at third trimester of pregnancy (T3) of pregnant women of the whole cohort, women with anemia at T3 (cases), and women without anemia at T3 (controls).

Clinical Characteristics and Biomarkers at T3	Whole Cohort	Women with Anemia at T3 (Cases)	Matched Women without Anemia at T3 (Controls)	*p*-Value
No. of women	629	128	128	
Systolic blood pressure, mm Hg, median (IQR)	120 (111–130)	120 (111–128)	121 (113–133)	0.094
Diastolic blood pressure, mm Hg, median (IQR)	75 (67–82)	74 (65–81)	77 (69–86)	**0.022**
Hypertension, *n* (%)				0.317
No	520 (97.7)	94 (95.9)	96 (98.0)	
Chronic isolated	1 (0.2)	0	0	
Gestational (without proteinuria)	1 (0.2)	0	0	
Moderate pre-eclampsia	8 (1.5)	3 (3.1)	2 (2.0)	
Severe pre-eclampsia	2 (0.4)	1 (1.0)	0	
Hb, g/L, median (IQR)	118 (111–126)	104 (100–107)	121 (115–130)	**<0.001**
MCV, fL, median (IQR)	87.0 (84.0–90.0)	84.0 (81.0–87.0)	88.0 (84.0–91.0)	**<0.001**
MCHC, pg, median (IQR)	29.4 (27.7–30.6)	27.6 (26.2–29.3)	29.7 (28.1–30.9)	**<0.001**
Term of delivery, weeks of amenorrhea, *n* (%)				0.876
<37	31 (5.3)	10 (8.5)	6 (5.1)	
37–41	453 (77.4)	90 (76.9)	92 (78.6)	
>41	101 (17.3)	17 (14.5)	19 (16.2)	
Birth weight of newborns, g, *n* (%)				0.157
<2500	41 (6.9)	9 (7.7)	7 (6.0)	
2500–3999	522 (88.2)	96 (82.0)	104 (88.9)	
≥4000	29 (4.9)	12 (10.3)	6 (5.1)	
Small for gestational age *, *n* (%)	37 (6.3)	5 (4.3)	4 (3.4)	1.000

Significant *p*-Values are reported in bold text (*p* < 0.05). T3: third trimester of pregnancy; IQR: interquartile range; Hb: hemoglobin value; MCV: mean corpuscular volume; MCHC: mean corpuscular hemoglobin concentration. * Birth weight below the 10th percentile according to gestational age and sex.

**Table 3:** Biomarkers at first trimester of pregnancy (T1) of pregnant women of the whole cohort, women with anemia at third trimester of pregnancy (T3) (cases) and women without anemia at T3 (controls).

Biomarkers at T1	Whole Cohort	Women with Anemia at T3 (cases)	Matched Women without Anemia at T3 (controls)	*p*-Value
No. of women	629	128	128	
Hb, g/L	127 (120–133)	123 (117–129)	129 (124–135)	**<0.001**
MCV, fL	86.0 (83.3–88.5)	85.5 (82.5–87.8)	86.3 (83.4–89.2)	**0.049**
MCHC, pg	29.8 (28.7–30.8)	29.4 (28.5–30.4)	30.1 (28.8–31.1)	**0.010**
RDW, %,	12.8 (12.3–13.2)	12.9 (12.3–13.4)	12.7 (12.4–13.2)	0.143
RHC, pg	34.1 (32.5–35.8)	33.9 (32.1–35.4)	34.1 (32.5–36.2)	**0.030**
SF, µg/L	-	27.0 (11.9–49.9)	43.6 (25.1–70.3)	**<0.001**
Hepc, µg/L	-	15.6 (3.8–24.4)	18.4 (10.9–24.0)	0.089
STfR, nmol/L	-	14.1 (12.2–17.4)	13.5 (12.0–15.3)	**0.030**
Ratio Hepc/SF	-	0.32 (0.18–0.67)	0.33 (0.20–0.53)	0.314
Ratio SF/STfR	-	1.9 (0.8–3.9)	3.3 (1.7–5.7)	**<0.001**

Biomarker data are presented as median (interquartile range). Significant *p*-Values are reported in bold text (*p* < 0.05). T1: first trimester of pregnancy; T3: third trimester of pregnancy; Hb: hemoglobin value; MCV: mean corpuscular volume; MCHC: mean corpuscular hemoglobin concentration; RDW: red cell distribution width. RHC = reticulocyte hemoglobin content; SF = serum ferritin; Hepc = serum hepcidin; STfR = serum transferrin receptor.

**Table 4:** Multivariate models of predictive biomarkers of anemia at third trimester of pregnancy (T3) in the matched nested case–control sample, using conditional logistic regressions.

Model	Biological Factors at T1	Unadjusted Standardized		Adjusted Standardized *			
				Without Selection Method		With Forward Selection Method	
		OR	*p*-Value	OR	*p*-Value	OR	*p*-Value
Model 1	Hb	0.63	<0.001	0.56	<0.001	0.66	<0.001
	MCV	0.85	0.043	0.70	0.128		
	MCHC	0.85	0.025	1.39	0.133		
	RDW	1.12	0.099	0.75	0.027		
	RHC	0.83	0.017	0.79	0.134		
	SF	0.76	0.002	0.73	0.009		
	Hepc	0.87	0.061	1.28	0.083		
	STfR	1.18	0.032	1.38	0.031		
Model 2	Hb	-	-	0.61	<0.001	0.66	<0.001
	MCV	-	-	0.70	0.118		
	MCHC	-	-	1.27	0.236		
	RDW	-	-	0.83	0.094		
	RHC	-	-	0.80	0.151		
	Hepc	-	-	1.15	0.257		
	Ratio SF/STfR	0.77	0.002	0.73	0.010		

T1: first trimester of pregnancy; OR: odds ratio; CI: confidence interval; Hb: hemoglobin value; MCV: mean corpuscular volume; MCHC: mean corpuscular hemoglobin concentration; RDW: red cell distribution width. RHC = reticulocyte hemoglobin content; SF = serum ferritin; Hepc = serum hepcidin; STfR = serum transferrin receptor. * Adjusted for parity, education level and the variables listed in the model.

## Data Availability

The data presented in this study are available on request from the corresponding author. The data are not publicly available due to privacy.
